# Factor XIII is a newly identified binding partner for platelet collagen receptor GPVI‐dimer—An interaction that may modulate fibrin crosslinking

**DOI:** 10.1002/rth2.12697

**Published:** 2022-04-24

**Authors:** Masaaki Moroi, Isuru Induruwa, Richard W. Farndale, Stephanie M. Jung

**Affiliations:** ^1^ Department of Biochemistry University of Cambridge Cambridge UK; ^2^ Department of Clinical Neurosciences University of Cambridge Cambridge UK

**Keywords:** crosslinking, factor XIII, fibrin, fibrin clot, GPVI, GPVI‐dimer, γ‐chain, γ‐dimer

## Abstract

**Background:**

In the fibrin‐forming process, thrombin cleaves fibrinogen to fibrin, which form fibrils and then fibers, producing a gel‐like clot. Thrombin also activates coagulation factor XIII (FXIII), which crosslinks fibrin γ‐chains and α‐chains, stabilizing the clot. Many proteins bind to fibrin, including FXIII, an established regulation of clot structure, and platelet glycoprotein VI (GPVI), whose contribution to clot function is largely unknown. FXIII is present in plasma, but the abundant FXIII in platelet cytosol becomes exposed to the surface of strongly activated platelets.

**Objectives:**

We determined if GPVI interacts with FXIII and how this might modulate clot formation.

**Methods:**

We measured interactions between recombinant proteins of the GPVI extracellular domain: GPVI‐dimer (GPVI‐Fc_2_) or monomer (GPVI_ex_) and FXIII proteins (nonactivated and thrombin‐activated FXIII, FXIII subunits A and B) by ELISA. Binding to fibrin clots and fibrin γ‐chain crosslinking were analyzed by immunoblotting.

**Results:**

GPVI‐dimer, but not GPVI‐monomer, bound to FXIII. GPVI‐dimer selectively bound to the FXIII A‐subunit, but not to the B‐subunit, an interaction that was decreased or abrogated by the GPVI‐dimer–specific antibody mFab‐F. The GPVI‐dimer–FXIII interaction decreased the extent of γ‐chain crosslinking, indicating a role in the regulation of clot formation.

**Conclusions:**

This is the first report of the specific interaction between GPVI‐dimer and the A‐subunit of FXIII, as determined in an in vitro system with defined components. GPVI‐dimer–FXIII binding was inhibitory toward FXIII‐catalyzed crosslinking of fibrin γ‐chains in fibrin clots. This raises the possibility that GPVI‐dimer may negatively modulate fibrin crosslinking induced by FXIII, lessening clot stability.

**Essentials**

- Platelet receptor glycoprotein VI (GPVI)‐dimer binds to collagen and fibrin.
- GPVI‐dimers, but not monomers, specifically bind to coagulation factor XIII (FXIII).
- GPVI‐dimer binds to FXIII via the A‐subunit and binds noncompetitively with FXIII to fibrin clots.
- GPVI‐dimers decrease fibrin γ‐chain crosslinking by FXIII and may modulate clot stability.

## 1 INTRODUCTION

Platelet collagen‐receptor glycoprotein VI (GPVI) occurs as both monomers and constitutively present homodimers, the functional form.^1^, ^2^ GPVI‐dimers bind to exposed subendothelial collagen fibers in injured vessels, initiating a signaling cascade leading to platelet activation, aggregate formation, and thrombus formation. GPVI‐dimer level is increased in activated platelets,^2^ and GPVI‐dimer clustering in activated platelets brings associated signaling molecules in closer proximity, enhancing signaling.^3^ Activated platelets present a membrane surface complex that stimulates the coagulation reaction, increasing active thrombin production.^4^ These multiple processes culminate in formation of a platelet thrombus,^5^, ^6^ which is stabilized by fibrin production due to thrombin generation on the surface of activated platelets.

GPVI has also been suggested to be a fibrin(ogen) receptor, and fibrin fiber formation from fibrinogen enhances collagen‐induced platelet activation.^7^, ^8^ Whether GPVI can actually bind fibrinogen remains controversial.^9^, ^10^ We recently demonstrated that GPVI‐dimers, not ‐monomers, bind to fibrin fibers in clots.^9^ Both collagen‐initiated platelet activation and coagulation pathways are necessary to form a stable blood clot, comprising many platelets, fibrin fibers, and coagulation factors, ensuring effective hemostasis and wound repair. Clot retraction which contributes to wound closure depends on the integrin α_IIb_β_3_‐fibrin interaction, which allows the platelet cytoskeleton to draw fibrin fibers together.^11^ Coagulation factor XIII (FXIII)^12^ was also reported to be involved in clot retraction,^13^, ^14^ although the mechanisms involved are yet not well defined.

Glycoprotein VI also binds to other proteins, including laminin,^15^ adiponectin,^16^ and extracellular matrix metalloproteinase inducer,^17^ so during the course of our studies on the GPVI‐fibrin interaction, we explored whether it interacts with FXIII, part of the plasma milieu in which thrombus formation occurs.

Plasma FXIII is an inactive tetramer of two A‐ and two B‐subunits (FXIIIA_2_B_2_), until it is cleaved by thrombin in the presence of calcium, which removes the activation peptide from subunit A, freeing it from the complex, and converting it to the active form (FXIIIAa, where "a" designates an active form) that functions as a transglutaminase.^18^ Platelet cytosol also contains abundant FXIII as active FXIIIA_2_, which is further activated by thrombin to FXIIIAa. Platelet FXIII was reported to be involved in clot retraction, platelet spreading and adhesion (as reviewed in Muszbek et al.^18^); and platelet FXIII was shown to crosslink several proteins to the platelet cytoskeleton, suggesting that it may be involved in platelet morphological change.^19^ However, FXIII deficiency does not affect platelet aggregation, indicating that it does not contribute to this process.^14^ Although FXIII is not exposed on the surface of resting platelets, it is surface expressed upon strong platelet activation.^12^ FXIIIAa crosslinks fibrin γ‐chains, forming γ‐dimers (γ‐γ), and further crosslinks γ‐ and α‐chains, forming higher‐molecular‐weight products. Crosslinked fibrin clots would be more stable and more resistant to fibrinolysis.

Platelets have been shown to participate in numerous coagulation processes, including fibrin formation, and therefore in this study we asked whether fibrin‐binding GPVI‐dimer may interact with FXIII, found in both platelets and plasma. We demonstrate that GPVI‐dimers specifically and directly interact with FXIII, and its inhibitory effect on γ‐γ fibrin crosslinking suggests that this interaction may modulate fibrin clot integrity.

## 2 MATERIALS AND METHODS

### 2.1 Materials

Table 1 shows the FXIII proteins (and their abbreviations) used in this study and their affinity constants (K_D_) for binding to GPVI‐dimer.

**TABLE 1:** Human FXIII proteins and their affinities to GPVI‐dimer

Material (abbreviations)	Details	K_D_ (μM)
Plasma FXIII^a^ (FXIIIA_2_B_2_)	Tetrameric, not activated	0.216 ± 0.067
FXIII A‐subunit^b^ (rh‐FXIIIA_2_)	Recombinant (rh) Not fully activated form	0.366 ± 0.047
FXIII A‐subunit (activated)^b^ (rh‐FXIIIAa)	Recombinant (rh) Thrombin‐activated form of rh‐FXIIIA_2_	0.140 ± 0.011
FXIII B‐subunit^b^ (rh‐FXIIIB_2_)	Recombinant (rh)	Too low to be determined

Note

**FIGURE 1:**
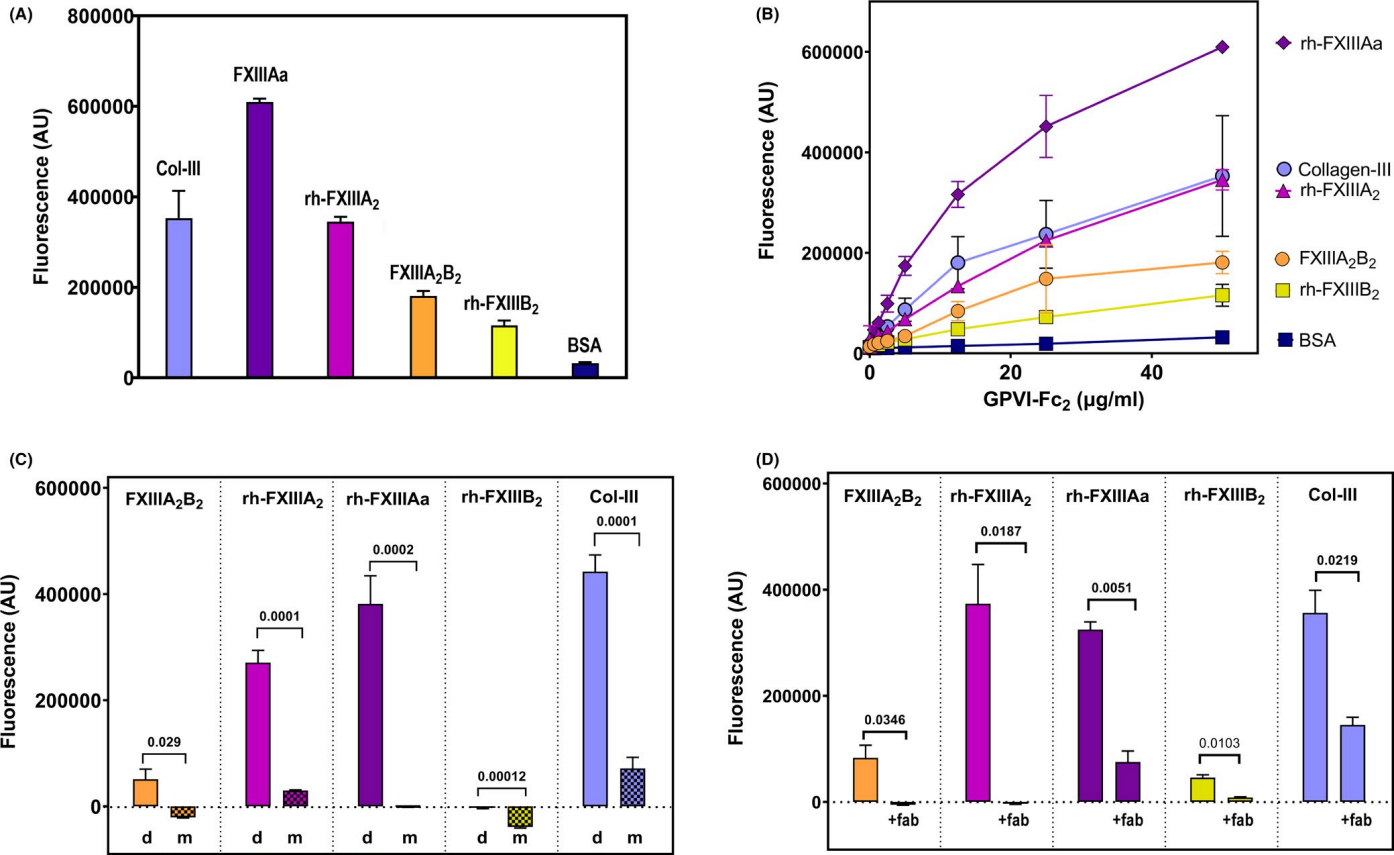
Analysis of GPVI binding to FXIII by ELISA. GPVI‐Fc_2_ binding to factor XIII proteins FXIIIA_2_B_2_, rh‐FXIIIAa, rh‐FXIIIA_2_, rh‐FXIIIB_2_ and the positive control collagen type‐III were determined by ELISA. The binding of GPVI‐Fc_2_ to FXIIIs were detected by anti‐GPVI antibody 1G5/ IR Dye 800CW anti‐mouse antibody. (A) Relative binding of GPVI‐Fc_2_ (50 µg/mL) to FXIII proteins coated at 10 µg/mL. The binding is expressed as fluorescence strength (arbitrary units, AU). (B) Concentration‐dependence of GPVI‐Fc_2_ binding. FXIIIA_2_B_2_ (orange ●), rh‐FXIIIA_2_ (pink ▲), rh‐FXIIIAa (purple ♦); rh‐FXIIIB_2_ (chartreuse □); collagen‐III (blue o); BSA (navy ■). The obtained K_D_s are described in Table 1. (C) GPVI‐Fc_2_ (GPVI‐dimer) and GPVI_ex_ (GPVI‐monomer) binding to FXIII proteins, with collagen‐III as a positive control. GPVI‐Fc_2_ or GPVI_ex_ was 50 µg/mL and the wells were coated with a FXIII protein or collagen type III (Col‐III) at 10 μg/mL. The *P* value (n = 3) comparing GPVI‐Fc_2_ binding to GPVI_ex_ binding for each FXIII protein is shown above each set of bars. GPVI‐Fc_2_ bound to col‐III and all FXIII proteins except for FXIII B‐subunit. GPVI_ex_ did not bind to any of the FXIII proteins and only weakly bound to col‐III. (D) Effects of GPVI‐dimer−specific antibody (mFab‐F) on GPVI‐Fc_2_ binding to FXIIIA_2_B_2_, rh‐FXIIIA_2_, rh‐FXIIIAa (thrombin activated rh‐FXIIIA_2_), rh‐FXIIIB_2_, and col‐III. The *P* value (n = 4) comparing no mFab‐F addition and addition of mFab‐F (+fab) of each FXIII protein is shown above each set of bars. The addition of mFab‐F (200 µg/mL) dramatically decreased or abrogated the binding of GPVI‐Fc_2_ to all the FXIIIs. FVIII, factor VIII; GPVI, glycoprotein VI

The FXIII proteins were obtained from the following suppliers: ^a^Fisher Scientific, Leicestershire, UK; and ^b^Zedira, Darmstadt, Germany. Binding affinities (K_D_) were calculated from the binding curves shown in Figure 1B; data are expressed as values of the mean ± SEM (n = 8). The binding affinity of the fully activated form of the FXIII A‐subunit is comparable to GPVI‐dimer’s affinity to collagen type III (0.183 ± 0.026).

Abbreviations: FVIII, factor VIII; GPVI, glycoprotein VI; SEM, standard error of the mean.

PK1 fibrinogen (FXIII‐, plasminogen‐, fibronectin‐free; Enzyme Research Laboratories, UK); recombinant proteins of human GPVI‐extracellular domain: GPVI‐Fc_2_ (dimer) and GPVI_ex_ (monomer), developed by Moroi and Jung, were previously described.^9^ The following antibodies were used: rabbit monoclonal antibody against human FXIIIa (EPR1360) and rabbit polyclonal anti‐fibrinogen γ‐chain antibody (AB96532) (Abcam, Cambridge, England); 1G5 (mouse monoclonal anti‐GPVI; Biocytex, Marseille, France); AlexaFluor‐647–conjugated streptavidin (Jackson Immunoresearch Laboratories, West Grove, PA, USA); and IRDye 800CW anti‐human antibody, IRDye 800CW anti‐rabbit antibody, and IRDye 680RD anti‐rabbit antibody (Li‐Cor, Lincoln, NE, USA).

### 2.2 ELISA to measure GPVI‐Fc_2_ binding to FXIII proteins

FXIII proteins, collagen (positive control), or bovine serum albumin (BSA; negative control) was incubated with ELISA plate wells (Nunc MaxiSorp, Thermo Fisher Scientific, Waltham, MA, USA) at 10 µg/mL (overnight, 4°C), and blocked with 0.5% BSA. The prepared wells were used for GPVI‐Fc_2_ (GPVI‐dimer) and GPVI_ex_ (GPVI‐monomer) binding assays as previously described.^19^ Bound GPVI‐Fc_2_ and GPVI_ex_ were detected by 1G5/IRDye 800CW anti‐mouse antibody and quantified using an Odyssey CLx fluorescence imaging system (Li‐Cor).

### 2.3 Immunoblotting analysis by clot assay

GPVI‐Fc_2_ and FXIII binding to fibrin fibers and its effects on fibrin crosslinking were analyzed using a fibrin clot assay.^9^ Figure 3A shows a schematic of a typical clot assay experiment. Each reaction mixture containing PK1‐fibrinogen, GPVI‐Fc_2_, 2 mM Ca^2+^, and FXIIIA_2_B_2_ was clotted by adding thrombin (1 U/mL). Each clot was isolated, washed thoroughly, dissolved in 8 M urea/2% SDS/5 mM 2‐mercaptoethanol and subjected to SDS‐PAGE/western blotting. To detect GPVI‐Fc and FXIII, respectively, the same blot was stained with IRDye 800CW anti‐human antibody and rabbit anti‐FXIII/IRDye 680RD anti‐rabbit antibody; band fluorescence was quantified following fluorescence imaging (Odyssey, Li‐Cor) and normalized to the respective protein band in the original unclotted sample and expressed as a percentage. On a separate blot of the same clot sample, the amounts of fibrin γ‐chain and γ‐dimer (γ‐γ) were detected by rabbit polyclonal anti‐fibrinogen γ‐chain antibody/IRDye 800CW anti‐rabbit antibody and expressed as a percentage of the γ‐chain band in the unclotted sample.

To determine if FXIII crosslinks GPVI‐Fc_2_ to other proteins, GPVI‐Fc_2_ (50 µg/mL), FXIIIA_2_B_2_ (0, 2, or 100 µg/mL), 2 mM Ca^2+^, and thrombin (1 U/mL), in the presence or absence of PK1‐fibrinogen, were reacted for 1 hour at 37°C. Some samples contained biotinamidopentylamine (1 mM; Sigma‐Aldrich, St. Louis, MO, USA), to incorporate biotin into substrates of FXIII. Immunoblotted membranes were stained by IRDye 800CW anti‐human antibody (Li‐Cor) for GPVI‐Fc and AlexaFluor‐647–conjugated streptavidin for biotin‐incorporated bands and visualized by fluorescence imaging (Odyssey, Li‐Cor).

## 3 RESULTS AND DISCUSSION

### 3.1 GPVI‐Fc_2_ specifically binds to FXIII

GPVI‐Fc_2_ binds with higher affinity to crosslinked fibrin than to noncrosslinked fibrin,^9^ suggesting that FXIII may affect its binding or crosslinked fibrin would show a higher affinity to GPVI‐Fc_2_. An ELISA assay was used to determine whether GPVI‐Fc_2_ and FXIII proteins are capable of direct interaction, as shown in Figure 1A (relative levels of binding at fixed GPVI‐Fc_2_ concentration) and Figure 1B (concentration‐dependency curves for calculation of K_D_ (dissociation constant) values summarized in Table 1). rh‐FXIIIAa (0.140 ± 0.011 µM) had the highest affinity, which was similar to the K_D_ for collagen type‐III (0.183 ± 0.026 µM). Although subunit B (rh‐FXIIIB_2_) shows some binding to GPVI‐Fc_2_ at 50 μg/mL (Figure 1A, chartreuse bar), its concentration‐dependency curve (Figure 1B, chartreuse squares) indicates that it scarcely binds to GPVI‐Fc_2_, having a nearly linear binding curve, reminiscent of a nonspecific interaction, precluding calculation of K_D_. These data indicate that the activated form of subunit A binds most strongly to GPVI‐Fc_2_. In contrast, GPVI‐monomer (GPVI_ex_) does not bind to any form of FXIII (Figure 1C) and binds only weakly to collagen‐III, indicating that GPVI must be in its dimeric form to bind to FXIII.

### 3.2 FXIII binding to GPVI‐Fc_2_ abrogated by inhibitory GPVI‐dimer–specific antibody

Figure 1D shows that although the binding of GPVI‐Fc_2_ to collagen is moderately inhibited by high concentration of the GPVI‐dimer–specific antibody mFab‐F (200 µg/mL), this concentration severely decreases or abrogates GPVI‐Fc_2_ binding to all the FXIII‐related proteins. This suggests that FXIII may bind near the collagen‐binding site of GPVI‐Fc_2_ since GPVI‐Fc_2_, not GPVI_ex_, binds specifically to collagen fibers^2^ and its binding to FXIII is also dimer‐ specific (Figure 1C). Addition of ZED A108, FXIII‐transglutaminase inhibitor (Zedira, Darmstadt, Germany), had no effect on GPVI‐dimer binding to FXIII (data not shown), showing that the transglutaminase activity of FXIII is not involved in the binding interaction, suggesting that GPVI‐dimer–bound FXIII may retain enzymatic activity for a small substrate like biotinamidopentylamine.

Our data indicate that the active form of GPVI, GPVI‐dimer, exclusively binds to FXIII A‐subunit and complex FXIIIA_2_B_2_, while showing little interaction with subunit B. Notably, FXIII subunit B, not subunit A, was reported to bind to fibrinogen and stimulate activation of FXIII on fibrin.^21^ This means that platelet FXIIIA_2_ could bind to GPVI‐dimers since it is exposed on the cell surface of strongly activated platelets.^22^, ^23^ Mitchell et al^23^ showed that platelet FXIII‐induced fibrin crosslinking occurs when no plasma FXIII is present, suggesting that active FXIII is exposed on the platelet surface, performing its function to crosslink fibrin chains. FXIII of activated platelets localizes in sphingomyelin‐rich rafts,^13^ and GPVI was also present in rafts on the platelet membrane.^24^ This raises the tantalizing possibility that GPVI‐dimer on the platelet membrane may serve as a receptor for platelet FXIII. Since the A‐subunit is an active subunit, the interaction with GPVI might also influence the transglutaminase activity of FXIII from plasma or platelets, modulating the crosslinking of fibrin clots.

### 3.3 GPVI is not a principal substrate of FXIII

**FIGURE 2:**
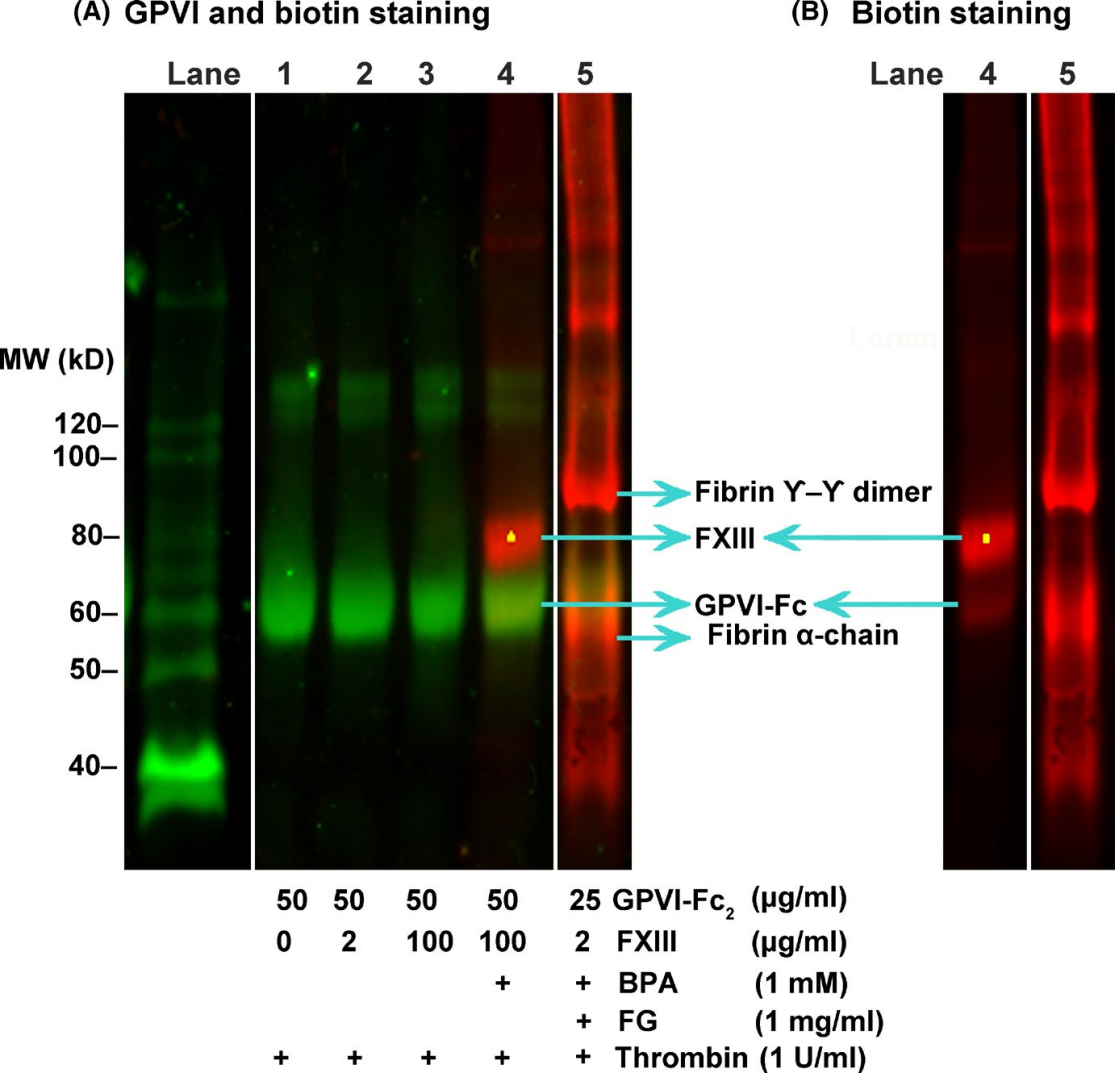
Analysis of factor XIII enzymatic activity. (A) In lanes 1‐4 (specific components in the reaction are given in the table below the blot), thrombin (1 U/mL) was added to a mixture of GPVI‐Fc_2_ (50 µg/mL) and FXIIIA_2_B_2_ and then allowed to react for 1 hour at 37°C. The samples were then analyzed by immunoblotting. In lane 5, GPVI‐Fc_2_ (25 µg/mL) and FXIIIA_2_B_2_ (2 µg/mL) is reacted in the presence of FXIII‐free fibrinogen (1 mg/mL) and the formed clot is isolated and similarly analyzed by immunoblotting. In addition, the reactions in lanes 4 and 5 are reacted in the presence of biotinamidopentylamine (1 mM) and incorporated biotin is detected by AlexaFluor 647–conjugated streptavidin (red). In this blot, GPVI‐Fc is detected by IRDye800CW anti‐human antibody (green). (B) Lanes 4 and 5 of the same western blot as panel A, showing only the biotin incorporation (AlexaFluor 647–conjugated streptavidin staining); the other lanes do not show any red staining. FVIII, factor VIII; GPVI, glycoprotein VI

It can be hypothesized that FXIII might crosslink GPVI to fibrin since GPVI binds more to crosslinked fibrin than to noncrosslinked fibrin.^9^ We tested this using reaction mixtures comprising GPVI‐Fc_2_, FXIIIA_2_B_2_, thrombin (1 U/mL), 2 mM Ca^2+^, and biotinamidopentylamine in the presence or absence of FXIII‐free fibrinogen and determined if the transglutaminase activity of FXIII can transfer the biotinyl moiety from biotinamidopentylamine to GPVI‐dimer, which would indicate that GPVI‐dimer is a substrate of FXIII. Figure 2A shows a western blot simultaneously stained for GPVI‐Fc (green) and proteins with incorporated biotin (red). In the sample with a high FXIII concentration (100 µg/mL; lane 4, Figure 2A), an obvious biotinylated protein band is seen at ≈80 kDa, and there is only a very faint band on the position of GPVI‐Fc that can be seen in Figure 2B (lane 4), which only shows the biotin staining. Since the molecular weights of FXIII A‐ and B‐subunit are 83 and 80 kD, respectively, the strong red band at around 80 kD suggests the incorporation of biotin to FXIII. Crosslinking of the FXIII A‐subunit to a fibrin clot was previously reported.^25^ The weak biotinylation of a protein that migrates at the GPVI position (lane 4, panel B) suggests some GPVI‐crosslinking may occur at high FXIII concentrations. In the fibrin clot, biotin was incorporated to fibrin α‐chain, γ‐γ dimer, and higher‐molecular‐weight bands, but little GPVI‐Fc (green) was associated with bands with molecular weight higher than GPVI‐Fc, suggesting that GPVI‐Fc would not be crosslinked to fibrin under our experimental conditions where FXIII is used at concentrations of 1–5 μg/mL. This figure indicates that crosslinking occurred predominantly in the fibrin γ‐ and α‐chains.

### 3.4 GPVI‐dimer–FXIII interaction in fibrin clots

**FIGURE 3:**
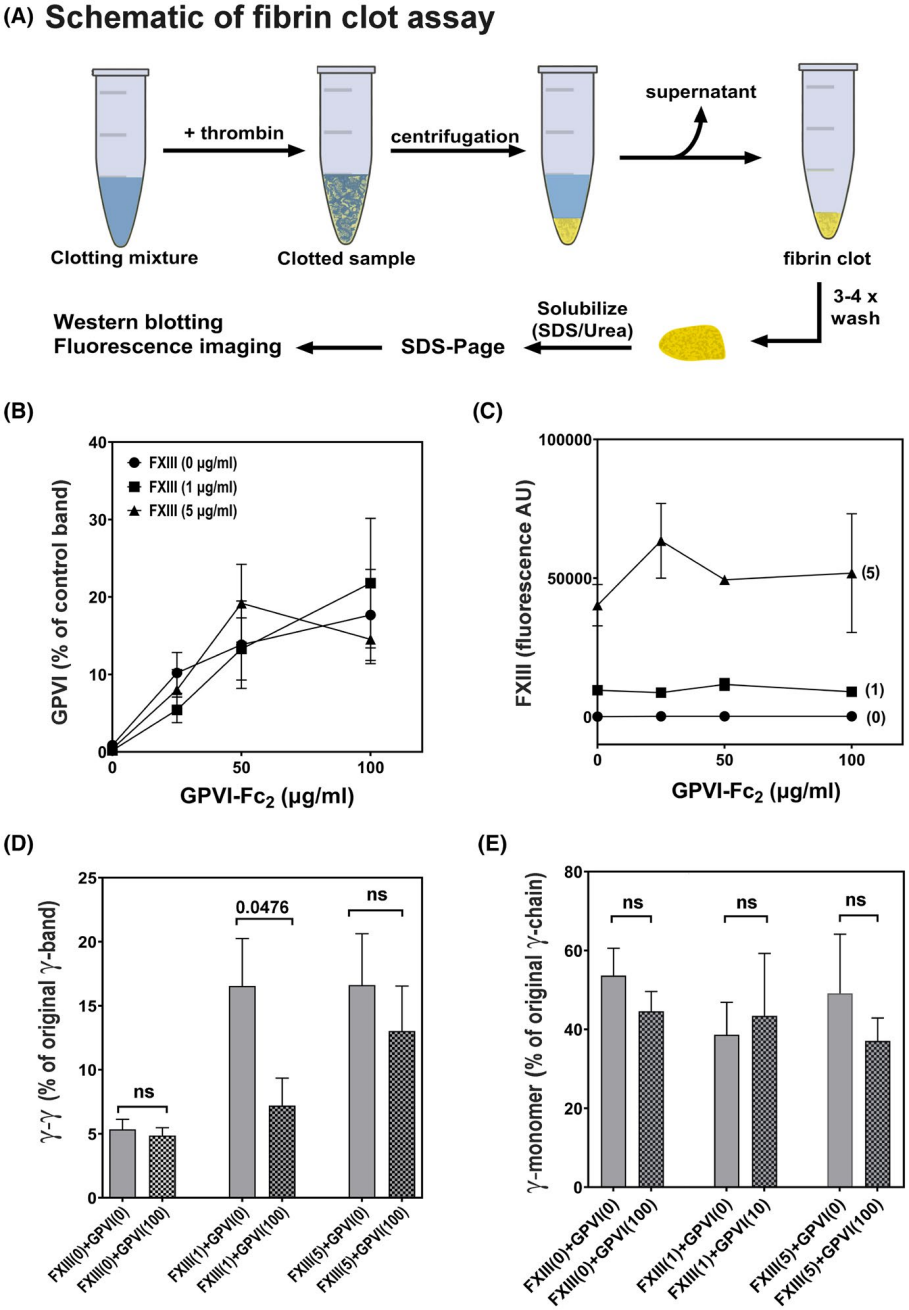
Effects of GPVI‐Fc_2_ on the binding of FXIII and on γ‐chain crosslinking. Each data value in all the graphs is the mean ± SEM (n = 4). (A) Schematic diagram of the fibrin clot assay, which we previously described in detail.^9^ In graphs (B) and (C), clots were formed from FXIII‐free fibrinogen, GPVI‐Fc_2_ (0, 25, 50, or 100 μg/mL), and FXIIIA_2_B_2_   at 0 (●), 1 (■), or 5 (▲) μg/mL and the binding of GPVI‐Fc to fibrin was determined. The amount of GPVI bound to fibrin (expressed as a percent of the control GPVI band (unclotted sample)) was not affected by FXIII. (C). The amount of FXIII bound to fibrin was not affected by GPVI‐Fc_2_. The fluorescence of the FXIII band was reported as arbitrary units (AU). (D) Amount of fibrin γ‐dimer (γ‐γ, expressed as % of the original γ‐band) in the clots formed in the presence of FXIIIA_2_B_2_ (0, 1, or 5 μg/mL) in the absence or presence of 100 μg/mL GPVI‐Fc_2_. In each two‐bar set, the checkered bars show the effect of adding GPVI‐Fc_2_ to FXIII at the indicated concentration. The clots formed in the presence of FXIIIA_2_B_2_ at 1 μg/mL show a significant decrease in γ‐dimer (*P* = .0476). (E) Amount of γ‐monomer (expressed as a percentage of the original γ‐band) in the clots formed in the presence of FXIIIA_2_B_2_ (0, 1, or 5 μg/mL) in the absence or presence of 100 μg/mL GPVI‐Fc_2_. In each two‐bar set, the checkered bars show the effect of adding GPVI‐Fc_2_ to FXIII at the indicated concentration. Adding GPVI‐Fc_2_ had no significant effect on the amount of γ‐monomer at any FXIIIA_2_B_2_ concentration. FVIII, factor VIII; GPVI, glycoprotein VI; SEM, standard error of the mean

We determined if FXIII might affect GPVI‐dimer binding to fibrin fibers and vice versa using our clot binding assay, which determines fibrin‐bound proteins by western blotting (Figure 3A: assay schematic and detailed in ref. 9). FXIII‐free fibrinogen is converted to fibrin by thrombin under different concentrations of FXIIIA_2_B_2_ (0, 1, 5 µg/mL) and GPVI‐Fc_2_ (0, 25, 50, 100 µg/mL). FXIII and GPVI‐Fc_2_ bound to fibrin were detected by staining the western blots with anti‐Fc and anti‐FXIIIA antibodies, respectively. Figure 3B (fibrin‐bound GPVI‐Fc vs GPVI‐Fc_2_) shows that the dose‐dependent increase in amount of GPVI‐Fc bound to fibrin is not affected by the amount of FXIII added to the clotting mixture, and the amount of FXIII bound to fibrin does not change with the concentrations of GPVI‐Fc_2_ in the clots (Figure 3C, fibrin‐bound FXIII vs GPVI‐Fc_2_). In this experiment, fibrinogen is converted to fibrin and FXIIIA_2_B_2_ is also activated to FXIIIAa by thrombin. The anti‐FXIIIAa antibody employed is specific for FXIIIA_2_, so this result demonstrates the fibrin‐specific binding of FXIIIA_2_. Bymes et al^21^ proposed a model where the FXIII B‐subunit binds to fibrinogen, which stimulates FXIIIA_2_ binding to fibrin D‐domain after fibrin formation and FXIII activation. GPVI‐dimer also binds to fibrin(ogen) D‐domain.^20^ This suggests that both proteins bind to the fibrin D‐domain but as our results show, GPVI and FXIII independently interact with fibrin. However, since GPVI‐Fc_2_ and FXIII can bind to each other, they could be in close proximity on the fibrin fiber.

The effect of GPVI‐Fc_2_ on FXIII‐catalyzed crosslinking was determined by the fibrin clot assay and quantitating bands on the western blot stained by γ‐chain–specific antibody. On the blots, γ‐and γ‐γ were identified by their molecular weight and the amounts calculated as percent of the amount of γ‐chain in the original (nonclotted) sample. As shown in Figure 3D, adding FXIII at 1 μg/mL increases γ‐dimer, and addition of GPVI‐Fc_2_ decreases it (middle set of bars), but this effect was not evident at higher FXIII concentration (rightmost set of bars). Since the enzymatic activity of FXIII is not involved in the binding to GPVI‐Fc_2_, the inhibitory activity of GPVI‐Fc_2_ would be due to steric hinderance. In contrast, adding GPVI‐Fc_2_ had no significant effect on the amount of γ‐monomer (Figure 3E).

## 4 CONCLUSION

Using an in vitro system of defined components, we demonstrated for the first time that GPVI‐dimer specifically interacts with the FXIIIAa (activated A‐subunit of FXIII), and FXIII and GPVI‐dimer independently bind to fibrin at proximal sites in the fibrin D‐domain. This suggests that the binding site for FXIII on GPVI‐dimer is different from its fibrin‐binding site and the binding site for GPVI‐dimer on FXIIIA_2_ is different from its fibrin‐binding site. We can hypothesize that the specific binding of GPVI‐dimer to FXIIIAa demonstrated in our study may negatively modulate the crosslinking activity of FXIIIAa and thus affect clot stability. The occurrence of this interaction between GPVI‐dimer and FXIII in the physiological context, in plasma and/or FXIII expressed on the activated platelet, and its function must be assessed in future work.

## RELATIONSHIP DISCLOSURE

None of the authors have any conflicts of interests to report.

## AUTHOR CONTRIBUTIONS

SMJ and MM designed and performed experiments, provided reagents, analyzed data, wrote the manuscript, and made the figures. II designed and performed experiments and read and made suggestions about the manuscript. RWF critically read the manuscript and made suggestions.
